# “Hong Long” Lychee (*Litchi chinensis* Sonn.) Is the Optimal Pollinizer for the Main Lychee Cultivars in Israel

**DOI:** 10.3390/plants11151996

**Published:** 2022-07-31

**Authors:** Amir Raz, Martin Goldway, Gal Sapir, Raphael A. Stern

**Affiliations:** 1 MIGAL—Galilee Research Institute, P.O. Box 831, Kiryat Shmona 11016, Israel; amirr@migal.org.il (A.R.); goldway@migal.org.il (M.G.); gal.sapir@edetepta.com (G.S.); 2 Department of Biotechnology, Faculty of Life Sciences, Tel-Hai College, Upper Galilee 12210, Israel

**Keywords:** lychee (*Litchi chinensis* Sonn.), pollination, fertilization, fruit set, yield

## Abstract

The lychee fruit is in high demand worldwide. However, the yields of many cultivars are low, including the high-quality cultivars “Nuomici” (NMC) and ”Fei Zi Xiao” (FZX), which are very tasty and produce large fruit with a small seed, but tend to shed their fruitlets. In a previous work, we found that cross-hand pollination of “Mauritius” (MA) with pollen of another cultivar increased fruit set and reduced fruit-drop in comparison to self-hand pollination. In the current research, we aimed to identify the optimal pollen donor for three of the main cultivars grown in Israel: MA, FZX, and “Tamuz” (TA). We compared the effect of different pollinizers and found that the Vietnamese cultivar “Hong Long” (HL), which is becoming an important cultivar in Israel, was the optimal pollinizer for the three cultivars. In addition, we found that FZX and TA were not self-fertile under the Israeli environmental conditions since they tend to shed fruitlets that originated from self-fertilization. In contrast, MA is able to fertilize itself, although cross-pollination greatly increased its fruit number and size. We also identified a new PCR marker for lychee, M3, that enabled us to distinguish between self- and cross-fertilized FZX fruits pollinated by HL. Our results indicate that cross-pollination, particularly by HL, has beneficial effects on the production of lychee and it is especially important for cultivars that generate small seeds and tend to shed their fruitlets.

## 1. Introduction

Lychee (*Litchi chinensis* Sonn.) is very popular in international markets since it is one of the world’s finest exotic fruits [1]. However, in all lychee-growing countries, the yields of most cultivars are low, including China, which produces around 70% (2.5 million tons) of the world’s consumption [2]. Lychee belongs to *Sapindaceae* and its flowering pattern displays three types of flowers: a female flower (F) and two types of male flowers (M1 and M2). M1 flowers first, then F, and finally M2 [3]. This pattern promotes cross-pollination [3,4]; however, self-pollination can occur. Although in a single inflorescence the flowering time of each type does not overlap (preventing self-fertilization of a given flower), still, pollination between inflorescences on the same tree can occur since their flowering time is not synchronized [4,5,6,7]. Hence, mono-cultivar lychee orchards can bear yield as was described, for example, for MA in Israel [8] and South Africa [5], for “Wai Chee” (WC) in China [9] and for “Haak Yip” (HY) in Taiwan [10]. Yet, cross-pollination positively effects the yield and fruit size, as was first reported by Stern et al. [11] and Degani et al. [12] for MA and “Floridian” (FL) grown in Israel, indicating that one of the factors responsible for the low yields of lychee could be the pollen source. Later, this phenomenon was also shown in other lychee cultivars in other countries, for example, for “Wai Chee,” pollinized by FZX and MA, or “McLean’s Red” in South Africa [13]. Moreover, Chu et al. [14] reported that in Taiwan, self-pollinated “Nuomici” (NMC) generates fruit with shriveled seeds and its fruitlets exhibit a high tendency to drop, but when pollinated with “Haak Yip,” its fruits were larger and the yield increased. 

The aim of this study was to investigate the effect of different pollen donors on fruit size and yield of the main lychee cultivars currently grown in Israel. 

## 2. Materials and Methods 

### 2.1. Field Experiments

The experiments were conducted during the years 2016–2021 in mature lychee orchards located in the Galilee region in northern Israel (Table 1). The tree density in all orchards was 500 trees per ha (5 m between rows and 4 m between trees).

MA was the main cultivar examined, and usually was planted as a single cultivar (“solid block”), whereas the pollen donors were planted in a single distinct row, so that the effect of the distance from the pollen donor on MA yield parameters could be determined. At every selected distance, six trees similar in size and bloom were marked. Each tree served as a replicate. The rows in all orchards were always aligned in the north-south direction. In most cases there was a good overlap between the flowering times of the cultivars. In all orchards, honeybee (HB) hives were introduced at a density of 5 hives/ha and bumble bee (BB) hives in a density of 10 hives/ha [16].

For determination of the pollen donor effect, the pollen acceptors FZX (in 2020 and 2021) or TA (in 2020), together with one of the pollen donors, were covered with a net and a BB hive was inserted. Four replicates were examined for each pollen donor. 

### 2.2. Greenhouse Experiments

The experiments were carried out in two greenhouses of 0.05 hectare each. Each greenhouse contained two rows of 12 trees (4 m between rows and 3 m between trees—830 trees per ha). Greenhouse 1 contained only MA trees, whereas greenhouse 2 contained one row of MA and another of HL. At bloom, two BB hives were inserted into each greenhouse and the temperature during the flowering period was kept at 25 °C. Each MA tree served as a replicate (24 in greenhouse 1 and 12 in greenhouse 2).

### 2.3. Initial Fruit Set, Yield, and Fruit Weight 

During 2016 and 2017 at Almagor orchard, 60 inflorescences per distance were marked at full female bloom (10 inflorescences per tree × 6 trees per distance). Each inflorescence contained around 150 female flowers. Three weeks after full female bloom, between the first wave of fruitlet drop (abnormal flowers, un-pollinated flowers etc.) and the second wave, the fruitlets were counted and the initial fruit set was calculated for each distance from the pollinizer [8]. The yield per marked tree in all experiments was recorded at harvest. Fruit weight was recorded in the field experiments only during 2017 at Lavi orchard and in the greenhouse experiment. The weight of 100 randomly selected fruits from each tree (6 MA trees (replicates) in the row adjacent to each pollinizer) was determined.

### 2.4. DNA Purification

DNA was extracted from young leaves, endosperm, and immature cotyledons of 4–6-week-old fruitlets. The procedure was based on Doyle and Doyle [17]. Briefly, 700 µL of extraction buffer (2% (*w*/*v*) hexa-decyltrimethyl-ammonium bromide (CTAB) in 100 mM Tris-HCl pH 8, 20 mM ethylenediamine tetraacetic acid (EDTA) pH 8, 1.4 M NaCl (*w*/*v*), 1% (*v*/*v*) polyvinylpyrrolidone (PVP MW 40,000), and 1% (*w*/*v*) 2-mercaptoethanol) were added to each sample, which had been ground in liquid nitrogen with a mortar and pestle. This mixture was incubated for 30 min at 65 °C and mixed occasionally. After cooling to room temperature, two extractions were performed with 400 µL of a 24:1 (*v*/*v*) mix of chloroform:octanol. The DNA was precipitated with 1.0 mL of 95% (*v*/*v*) ethanol and dissolved in 50 µL double-distilled water.

### 2.5. Marker Identification by iPBS

Specific iPBS sites were identified by the method of Kalender et al. [18]. The best results were obtained using the 2074 primer (5′ GCTCTGATACCA 3′). Each 50 µL PCR reaction contained 25 µL of X2 HyTaq mix (HyLabs, Israel), 1 µL of 2074 primer (10 µM), and 1 µL DNA. The PCR program was 35 cycles of 95 °C for 15 s, 50 °C for 60 s, and 72 °C for 60 s. Reactions were analyzed by gel electroporation and specific bands were extracted from the gel, purified by NucleoSpin Gel and a PCR clean-up kit (MACHEREY-NAGEL, Germany), and sequenced. The resulting sequences were used to design specific primers for each band. For the M3 marker, which was identified in this work using the 2074 primer, the PCR reaction contained 10 µL of X2 HyTaq mix (HyLabs, Israel), 0.5 µL M3-fw primer (5′ TTGGATTCCTTCGAAGAGGCC 3′), 0.5 µL M3-rev primer (5′ TAACTTCCAAGACAAGCTAGCG 3′) at 10 µM, and 1 µL DNA in 20 µL reaction. The PCR program was 30 cycles of 95 °C for 15 s, 50 °C for 60 s, and 72 °C for 60 s. 

### 2.6. Statistical Analysis

Percentage data were subjected to Arcsin transformation before analysis to provide a normal distribution. Data were analyzed for statistical significance using the general linear model (GLM) procedure. Duncan’s new multiple range test was applied to compare treatments when ANOVA showed significant differences between the means at *p* ≤ 0.05.

## 3. Results

### 3.1. Effect of Different Pollinizers on the Yield of MA 

Almagor orchard: The experiment was carried out in 2016 and 2017. The effect on the fruit set and yield at increasing distances between the pollinizer HL and the pollen acceptor MA was examined. A positive effect of the distance from HL on the MA fruit set was observed as early as three weeks after bloom. As shown in Table 2, the MA fruit set rate in the adjacent row (6 m) to HL was 10–12%, whereas when HL was two rows away (12 m) the MA fruit set was reduced to 7–9% (not in all cases significant). A further reduction was observed three rows away from HL (18 m, not significant). In accordance, MA yields were 50–60 kg/tree when HL was one row away, 30–36 kg/tree when two rows away, and 20 kg/tree when three rows away (Table 2).

**Figure 1:**
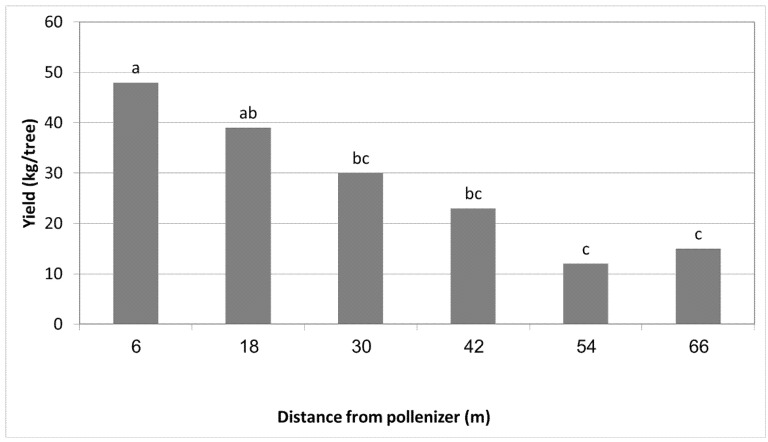
The effect of increasing distance from the pollinizer HL on the yield of MA, Ravid 2017. Mean values of each column (distance) followed by different letters differ significantly per Duncan’s new multiple range test, *p* < 0.05. Data of each column are the means of 6 replicate trees per treatment (distance).

Ravid orchard: The experiment was performed in 2017. The weather at Ravid orchard is cooler and more humid than at Almagor. Like in the experiment at Almagor, HL was the pollinizer of MA (Table 1). However, despite the different climate conditions, similarly to the experiment at Almagor, the yield of MA reduced as the distance from HL was increased: 48 kg/tree when HL was in the adjacent row, 40 kg/tree when HL was two rows away, 30 kg/tree when HL was three rows away, and 24 kg/tree when four rows away, namely, a reduction of 50% (Figure 1).

**Figure 2:**
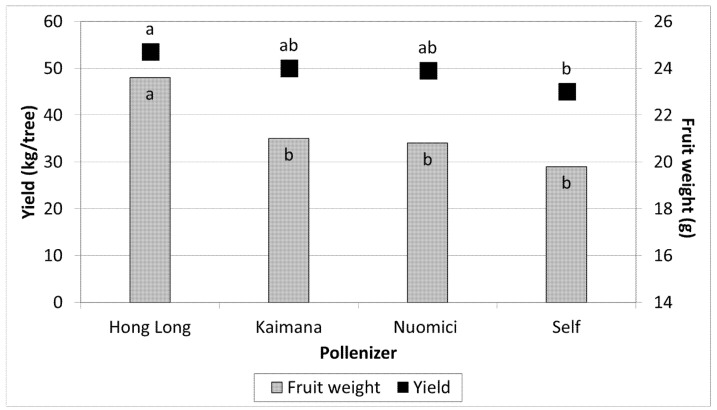
The effect on fruit weight and yield of MA adjacent to different pollinizers, Lavi 2017. Mean values of each column followed by different letters differ significantly per Duncan’s new multiple range test, *p* < 0.05. Data on yield are the means of 6 MA trees (6 replicates) adjacent to each pollinizer. Data of fruit weight are the mean of 100 randomly fruit from each of the 6 trees per treatment.

Lavi orchard: In 2017 three pollinizers for MA were compared: HL, KA, and NMC (Figure 2). The yield of MA in the closest row to HL was 48 kg/tree, whereas in the closest row of MA to KA or NMC it was 35 kg/tree, similar to the self-pollinated control. Moreover, MA fruit from trees adjacent to HL were larger and reached 24.5 g per fruit compared to only 21 g per fruit in the row adjacent to KA or NMC. Hence, not only was the yield higher (25 ton/ha), but the fruit weight also increased when the MA was pollinized by HL. 

Greenhouse experiment: In three consecutive years from 2018 to 2020 the impact of HL pollination on MA yields was examined in two greenhouses: Greenhouse 1 contained two rows of only MA trees and greenhouse 2 contained a row of MA and a row of HL. In all three years, MA trees pollinated by HL produced higher yield, reaching 35 kg/tree in comparison to 23 kg/tree in the self-pollinated MA in greenhouse 1 (Table 3). Despite the high fruit load, the average fruit weight was around 10% higher in the MA trees in greenhouse 2. This high yield resembles 30 ton/ha. 

### 3.2. Effect of Different Pollinizers on the Yield of FZX

**Figure 3:**
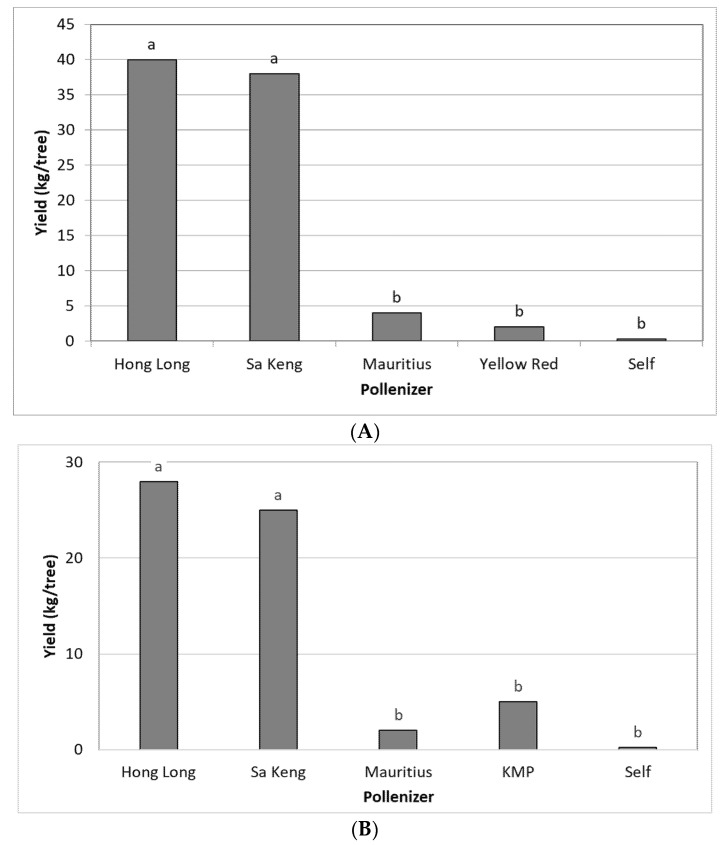
The effect of different pollinizers under nets on the yield of the adjacent FZX trees at Moran orchard in 2020 (**A**) and 2021 (**B**). Mean values of each column (pollinizer) followed by different letters differ significantly per Duncan’s new multiple range test, *p* < 0.05. Data on yield are the mean of 4 FZX trees (4 replicates).

The experiments were performed in 2020 and 2021 at Moran orchard. Five cultivars—SK, HL, MA, YR, and KMP—were compared as pollinizers of FZX. A single FZX tree together with a single tree of one of the five pollinizers was covered with a net and a BB hive was inserted under the net. No self-fertilization was observed in single FZX trees covered with nets. Thus, FZX fertilization depends totally on cross-pollination. Pollination with HL and SK resulted in a high yield of 30–40 kg/tree (15–20 ton/ha), whereas when pollinated with MA, YR, or KMP the yield was very low (Figure 3). 

### 3.3. Effect of Different Pollinators on the Yield of TA

**Figure 4:**
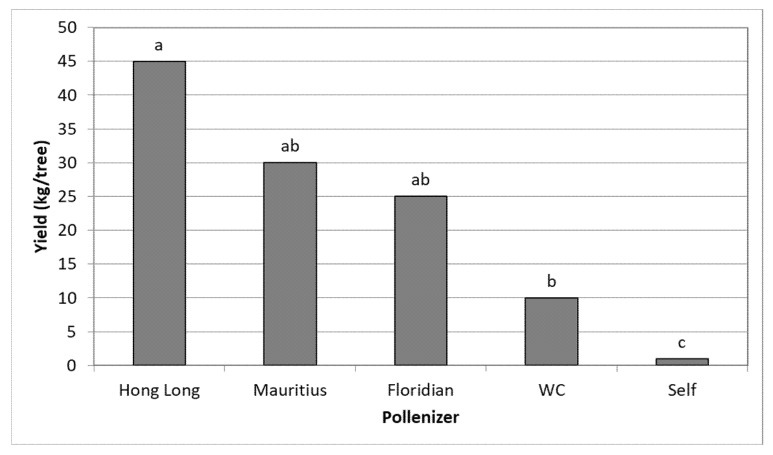
The effect of different pollinizers under nets on yield of the adjacent TA trees, Ravid 2020. Mean values of each column (pollinizer) followed by different letters differ significantly by Duncan’s new multiple range test, *p* < 0.05. Data on yield are the mean of 4 TA trees (4 replicates).

Ravid orchard: The experiment was carried out in 2020. This one-hectare orchard contains a solid block of TA with HL, MA, FL, and WC as single pollen-donor trees planted within the block. The orchard is flanked by an HL orchard in the east and an MA orchard in the west. Each pollen donor and an adjacent TA tree were covered with a net and a BB hive was inserted, and two adjacent TA trees covered with a single net served as the control. TA pollinated by HL provided the highest yield of 45 kg/tree (23 ton/ha); with pollination by FL and MA, TA yielded 25–30 kg/tree; and pollination by WC provided 10 kg/tree (Figure 4). The yield of self-pollinated TA was minor.

In the following year, 2021, due to low bloom of the pollen donors, the yield was measured on TA trees adjacent to the HL or the MA orchards, and trees at the center of the TA block (which were 50 m away from the HL or MA orchards) served as a control. Supporting the results of the previous year, TA adjacent to HL yielded 23 kg/tree, and next to MA 15 kg/tree. The control TA trees, which most likely were mainly self-pollinated, yielded only 6 kg/tree.

High yield of TA due to cross-pollination was supported by results from the Moran orchard. This 15 ha orchard contains TA and MA trees planted alternately along the rows. TA yield was measured on 20 trees in the central row, and it was 30 in 2020 and 24 kg/tree in 2021. 

### 3.4. Identifying Genetic Markers for Paternal Identification

**Figure 5:**
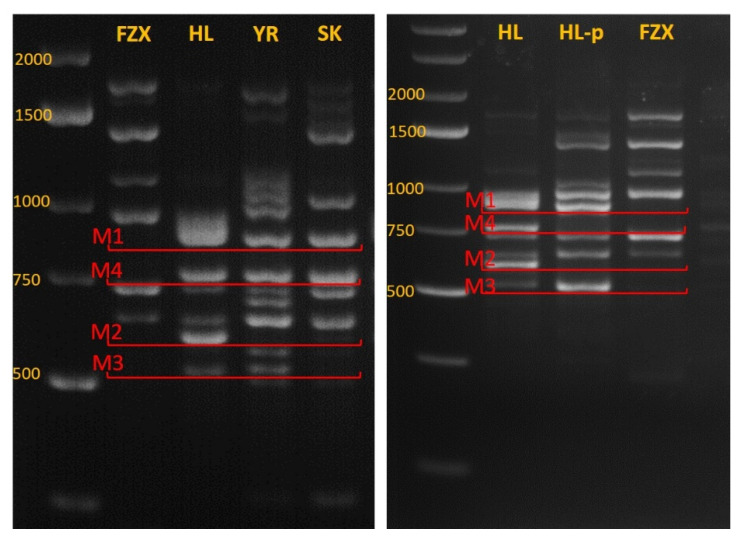
iPBS genetic fingerprints of different cultivars. Left—FZX and three potential pollinizers. Right—HL, HL-p = HL progeny, FZX.

We looked for a fast and simple way to identify PCR markers, which would help us to discriminate between self and cross-fertilization and between the different potential pollinizers. Since the genomic information at the time of the experiment was very poor (only lately was a first draft of the lychee genome published, by Hu et al. [19]) we used the Inter Primer Binding Site (iPBS) amplification method [18]. This method applies the conserved PBS site of LTR retrotransposons to amplify genomic sequences that are flanked by inverse repeats of LTRs. Since LTR retrotransposons are highly dispersed in many plants, the amplification yields a characteristic DNA “fingerprint.” By comparing the fingerprints of different cultivars, a distinctive band can be isolated and sequenced. The sequences are used to design cultivar-specific primers for PCR. We focused on defining self- and cross-fertilization of FZX by HL. The comparison between iPBS fingerprints of FZX and HL revealed four bands (M1–M4) that appeared in HL (and other pollinizers) but did not appear in FZX (Figure 5). However, two of them, M2 and M4, were not amplified in an HL progeny, indicating that HL is heterozygous for these markers (Figure 5). M1 and M3 products were sequenced and specific primers for each of them were designed to examine the presence of M1 and M3 in 30 HL progenies. M3 appeared in all of them, whereas M1 did not. Hence, the M3 marker served as a paternal marker for HL in FZX progenies. 

### 3.5. Genetic Analysis of FZX Fertilization by HL 

The M3 marker was used to analyze the rate of self- versus cross-fertilization in progenies of FZX trees planted adjacent to HL trees that were either under nets with BB hives or exposed to open pollination. Fruitlets were sampled six weeks post anthesis, when embryos were big enough to be isolated and before they degenerated. The fruitlet’s DNA was tested for the presence of M3 in the progenies (Table 4). In FZX trees, which were under nets (with no pollinizers), only a few flowers set fruits; however, none of them survived till harvest. FZX trees that were adjacent to HL trees, either under a net or exposed to open pollination, exhibited 53% and 54% of foreign fertilization, and high yields of 12 and 15 kg/tree, respectively. In the net treatment, cross-pollination was most probably from HL. As for the open pollination, MA trees also flowered nearby, and the M3 marker could not differentiate between HL and MA. However, in a few experiments we carried out in recent years we found that MA could not fertilize FZX trees, even in optimized conditions (Figure 3). Hence, the significant improvement in fruit sets and yields in the open pollination treatment was most likely due to HL fertilization. 

### 3.6. Fruitlet Genetic Analysis and Yield of FZX Fertilization by SK 

In a similar experiment, in a different plot of the Moran orchard, we examined the fruit-set ratio and yields of FZX trees planted adjacent to SK, either under nets or exposed to open pollination. Again, the M3 marker was used to identify cross-fertilization and the analysis was performed on six-week-old fruitlets before the embryo was degenerated. However, in SK, M3 is heterozygous and therefore was expected to appear in only half of the FZX fruitlets that were obtained as a result of fertilization by SK. We found that in the net experiment, 24% of the fruitlets contained M3, indicating that 48% of all fruitlets resulted from SK fertilization, whereas in open pollination 44% of the fruitlets contained M3, indicating that 88% of all fruitlets resulted from SK fertilization (Table 4). It should be noted that in addition to SK, MA also bloomed in the orchard; however, as in the HL experiment, we assumed that all cross-fertilization events of FZX resulted from SK pollination, since MA is a very poor pollinizer for FZX. 

**Figure 6:**
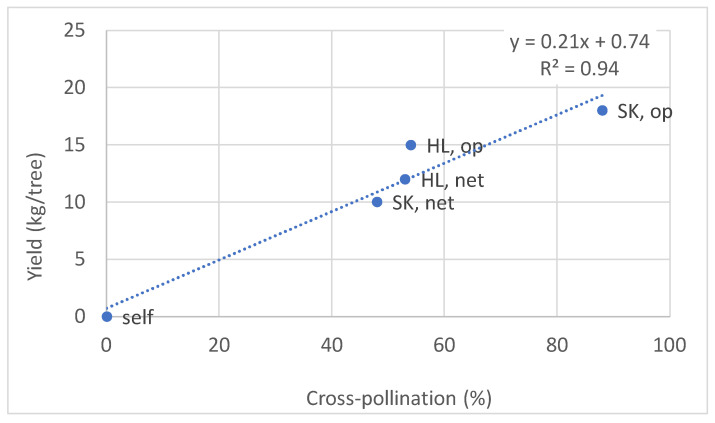
Correlation between the rate of cross-pollinated and final fruit yield. Op—open pollination. Self—self-pollinated under a net. Net—trees covered with nets. Each point is an average of 4 trees (replicates).

The yields of FZX correlate well with the cross-pollination rates (Figure 6); high in open pollination, moderate when FZX and SK were covered by the same net, and not when FZX was solely under the net. Although cross- versus self-fertilization was determined only in young fruitlets (since the seeds in mature fruits of FZX are degenerated), it is still reasonable to assume that the trend did not change significantly in the fruitlets that reached maturity. Self-fruitlets may tend to drop, as indicated by the very low yield of self-pollinated FZX. 

## 4. Discussion 

Increasing lychee yield is a challenge in Israel, as well as in other lychee-growing countries. It is well established in many cultivated fruits that pollen donors differ in their impact on the yield of the pollen acceptor. In Israel, MA is the main cultivar and FZX and TA are among the most important ones. However, defining the optimal pollinizer for these cultivars has not been accurately assessed and therefore orchards do not present their full yield potential.

We determined that FZX and TA are not self-fertile in the environmental conditions of Israel. Hence, FZX and TA are dependent on cross-pollination, whereas MA, although self-fertile, provides a higher yield when cross-pollinated [3,11,12]. Defining the optimal pollen donor for these cultivars revealed that the yield of TA was high when pollinated by both HL and MA, whereas FZX exhibited a high yield when pollinated by HL but low when pollinated by MA. As for MA, pollination by HL provided higher yield and larger fruit than when pollinated by KA or NMC. Thus, we found that HL is the optimal pollinizer for MA, TA, and FZX, exhibiting the importance of defining the best pollen donor for each lychee cultivar.

Why is HL so effective? HL blooms before MA, and a possible explanation is that it provides pollen from M2 flowers, which were shown to be more potent than M1 flowers [3,4,6,20]. However, this does not explain the competence of HL as a pollinizer of FZX and TA. A similar attribute was found for the “Etinger” avocado cultivar in Israel, which is the best pollinizer for many cultivars, including Hass [21,22,23,24]. The genetics and physiological aspects of this quality will be analyzed. 

Moreover, MA trees fertilized by HL exhibited not only higher yields but also larger fruits (Figure 2). The effect of genes from the male parent on the development of fruit or seeds is known as xenia. The pericarp and aril from outcrossed fruit are heavier than self-fertilized ones. Xenia has also been described for another few combinations of lychee cultivars [11,12,13,25].

Under Israeli climate conditions, self-pollinated FZX does not provide any yield, and although some fruitlets develop, they drop before maturity. Seed degeneration was also reported for other lychee cultivars such as NMC, Kwai-Mi-Red, and Gui-wei. These elite cultivars also present intensive fruit drops when self-pollinated and high survival rates when cross-pollinated [3,11,13,14,26]. Jiang et al. [27] found that FZX pollen has low viability and was not efficient in fertilizing itself or other cultivars. However, a few additional possible reasons could cause this outcome, such as specific rejection of the pollen, poor development of the seed [28], or early fruitlet drop [3,11,13,14,26]. Rejection of a specific pollen donner by a pollen acceptor is termed unilateral cross incompatibility (UCI). Recently, Wang et al. [29] reported UCI in longan (*Dimocarpus longan*), which belongs to the *Sapindaseae* family, as does lychee. The popular longan cultivar in Thailand, Yisuo, is self-fertile and can be fertilized by many cultivars but not by Shixia pollen, since in this cross, the pollen tube germinates in the stigma and through the style but fails to penetrate the egg cell. Similar results were reported by Cuevas and Polito (1997) [30], who studied the fertilization process of the “Manzanillo” olive in California with three different pollinizers—"Sevillano,” “Mission,” and “Ascolano.” They found that only “Sevillano” increased the fruit set and yield of the “Manzanillo” compared to self-fertilization, whereas the two others had no impact at all. It is possible that the cross between the FZX lychee flower and MA pollen suffers from the same phenomena. However, this hypothesis will need further investigation. 

Another aspect of this work was the identification of a molecular marker for accurate detection of cross- versus self-fertilization. Molecular markers have been developed for many crops, including trees, and are utilized for detecting the “pollen parent” among progenies [31,32,33,34]; however, for lychee, such markers have not been reported yet. Indeed, a few works in the past developed different genetic markers for cultivar identification, such as SSR, AFLP, and RAPD [35,36,37]. These methods resulted in genetic fingerprints, which comprise tens of different bands, and in many cases, their combinations are unique to each cultivar. However, considering that in fruit trees the heterozygosity of both pollen donor and acceptor is high and that some of these markers are homozygous while others are heterozygous, it may be challenging to rely on these markers for the identification of the “pollen parent.” Here, we detected a simple-to-use marker that could discriminate between FZX fruit obtained by self-fertilization or by cross-fertilization with HL or SK. This marker, M3, was obtained using the conserved PBS site of LTR retrotransposons, which are widely dispersed along the genome and hence may provide many more cultivar-specific markers. In addition, combining our method with the data from the recently published lychee genome [19] will greatly simplify the process of developing many more genetic markers for such purposes.

## 5. Conclusions 

Our results indicate that inclusion of cross-pollinating cultivars, especially HL, in a lychee orchard will have beneficial effects on the production of all cultivars examined in this work and probably additional cultivars, too, especially those with small seeds, which tend to abort and drop their fruits. 

## Figures and Tables

**Table 1:** Lychee plots that served for the experiments during the years 2016–2021.

Year	Orchard	Altitude (m)	Female cv. Tested	Pollinizer	Type of Pollination
2016	Almagor	−200	MA	HL	OP
2017	Almagor	−200	MA	HL	OP
	Ravid	+200	MA	HL	OP
	Lavi	+300	MA	HL, KA, NMC	OP
2018–2020	Orchard Farm	+100	MA	HL	Greenhouse
2020	Moran	+150	FZX	HL, SK, MA, YR	Net
	Ravid	+200	TA	HL, MA, WC, FL	Net
2021	Moran	+150	FZX	HL, MA, SK, KMP	Net
	Ravid	+200	TA	HL, MA	OP

The cultivars that were tested as females were “Mauritius” = MA, “Fei Zi Xiao”= FZX and “Tamuz” = TA (an Israeli cv., a progeny of “Kaimana” = KA × “Nuomici” = NMC from the Israeli breeding program, previously called BD 17-70, [15]). The pollinizers tested were “Hong Long” = HL (from Vietnam), “Sah Keng” = SK (from Taiwan), “Yellow Red” = YR (seedling of “Brewster” from Florida), “Wai Chee” = WC (“Huai Zhi” from China), NMC (from China), “Floridian” = FL (seedling of “Brewster” from Florida), “Kwai Mi Pink” = KMP (B#3 from Australia), and “Kaimana” = KA (seedling of “Haak Yip” = “Heiye” from Hawaii). The trees in each orchard were uniform in age (ca. 10 years) and size, and had similar crop loads in the year prior to the experiments.

**Table 2:** The effect of the pollinizer HL on fruit set and yield of MA trees at different distances, Almagor 2016–2017.

MA Row (no.)	Distance from HL (m)	2016		2017	
		Fruit Set (%)	Yield (kg/Tree)	Fruit Set (%)	Yield (kg/Tree)
1	6	10.3 a	52 a	12.5 a	62 a
2	12	7.0 b	36 b	9.3 ab	30 b
3	18	4.5 b	24 c	5.0 b	21 c
4	24	4.7 b	28 c	-	-
5	30	3.8 b	22 c	-	-
6	36	4.4 b	20 c	-	-

Mean values within a column followed by different letters differ significantly per Duncan’s new multiple range test, *p* < 0.05. Data of fruit percentage are the means of 60 inflorescences per distance (10 inflorescences per tree × 6 trees per distance). Data of yield are the mean of the same 6 trees per distance.

**Table 3:** The effect of the pollinizer HL on the yield and fruit weight of MA trees in the greenhouse, Orchard Farm 2018–2020.

Greenhouse	HL Pollinator (+/−)	MA Yield					
		2018		2019		2020	
		Yield (kg/tree)	Fruit Weight (g)	Yield (kg/tree)	Fruit Weight (g)	Yield (kg/tree)	Fruit Weight (g)
1	−	25 b	21.8 b	20 b	22.4 a	23 b	22.1 b
2	+	38 a	24.6 a	35 a	24.5 a	32 a	23.9 a

Mean values of each column followed by different letters differ significantly per Duncan’s new multiple range test, *p* < 0.05. Data on yield are the mean of 24 trees (replicates) in greenhouse no. 1 or 12 trees in greenhouse no. 2.

**Table 4:** Fertilization rate and final yields of FZX by different pollinizers (OP = open pollination).

Orchard Plot	Pollinizers	Cross-Fertilized Progenies (%)	Self-Fertilized Progenies (%)	Yield (kg/Tree)
East	FZX, self	0	100	0
East	HL, net	53	47	12
East	HL, op	54	46	15
West	SK, net	48	52	10
West	SK, op	88	12	18

Each treatment contains 4 trees; 30 fruitlets/tree.

## References

[1] Huang X., Subhadrabandhu S., Mitra S.K., Ben-Arie R., Stern R.A., Menzel C.M., Wait G.K. (2005). Origin, History, Production and Processing. Litchi and Longan: Botany, Cultivation and Uses.

[2] Huang X., Yahia E.M. (2019). Achieving Sustainable Cultivation of Litchi. Achieving Sustainable Cultivation of Tropical Fruit.

[3] Stern R.A., Gazit S., Janick J. (2003). The Reproductive Biology of the Lychee. Horticultural Reviews.

[4] Davenport T., Stern R.A., Menzel C.M., Waite G.K. (2005). Flowering. Litchi and Longan: Botany, Cultivation and Uses.

[5] Joubert A.J., Monselise S.P. (1986). Litchi. Handbook of Fruit Set and Development.

[6] Stern R.A., Gazit S. (1996). Lychee Pollination by the Honeybee. J. Am. Soc. Hortic. Sci..

[7] Osuna E.T., Valenzuela R.G., Muy R.D., Gardea B.A., Villarreal R.M. (2008). Sex Expression and Flower Anatomy of Litchi (*Litchi chinensis* Sonn.). Rev. Fitotec. Mex..

[8] Stern R.A., Kigel J., Tomer E., Gazit S. (1995). ‘Mauritius’ Lychee Fruit Development and Reduced Abscission after Treatment with the Auxin 2,4,5-TP. J. Am. Soc. Hortic. Sci..

[9] McConchie C.A., Batten D.J. Floral Biology and Fruit Set in Lychee. Proceedings of the 2nd National Lychee Symposium.

[10] Batten D.J., McConchie C.A. Pollination in Lychee. Proceedings of the Third National Lychee Seminar.

[11] Stern R.A., Gazit S., El Batsri R., Degani C. (1993). Pollen Parent Effect on Outcrossing Rate, Yield, and Fruit Characteristics of ‘Floridian’ and ‘Mauritius’ Lychee. J. Am. Soc. Hortic. Sci..

[12] Degani C., Stern R.A., El-Batsri R., Gazit S. (1995). Pollen Parent Effect on the Selective Abscission of ‘Mauritius’ and ‘Floridian’ Lychee Fruitlets. J. Am. Soc. Hortic. Sci..

[13] Froneman I.J., Bijzet Z., Sippel A.D., Bower J.P. (2012). Effect of Different Pollen Parents on Fruit Retention and Fruit Characteristics in ‘Wai Chee’ Litchi. Acta Hortic..

[14] Chu Y.C., Lin T.S., Chang J.C. (2015). Pollen Effects on Fruit Set, Seed Weight, and Shriveling of ‘73-S-20’ Litchi-with Special Reference to Artificial Induction of Parthenocarpy. HortScience.

[15] Goren M. (2013). The Advantage of Growing New Litchi Varieties. Alon Hanotea.

[16] Sapir G., Goldway M., Stern R.A. (2019). Supplementing Bumblebees to ‘Mauritius’ Lychee Improves Yield. Sci. Hortic..

[17] Doyle J.J., Doyle J.L. (1987). A Rapid DNA Isolation Procedure for Small Quantities of Fresh Leaf Tissue. Phytochem. Bull..

[18] Kalendar R., Antonius K., Smýkal P., Schulman A.H. (2010). IPBS: A Universal Method for DNA Fingerprinting and Retrotransposon Isolation. Theor. Appl. Genet..

[19] Hu G., Feng J., Xiang X., Wang J., Salojärvi J., Liu C., Wu Z., Zhang J., Liang X., Jiang Z. (2022). Two Divergent Haplotypes from a Highly Heterozygous Lychee Genome Suggest Independent Domestication Events for Early and Late-Maturing Cultivars. Nat. Genet..

[20] Stern R.A., Gazit S. (1998). Pollen Viability in Lychee. J. Am. Soc. Hortic. Sci..

[21] Gazit S., Gafni E. (1986). Effect of Hand Pollination with Different Pollen Donors on Initial Fruit-Set in Avocado. Isr. Agresearch.

[22] Degani C., Goldring A., Gazit S. (1989). Pollen Parent Effect on Outcrossing Rate in ‘Hass’ and ‘Fuerte’ Avocado Plots during Fruit Development. J. Amer. Soc. Hort. Sci.

[23] Gazit S., Degani C., Whiley A.W., Schaffer B., Wolstenholme B.N. (2002). Reproductive Biology. The Avocado: Botany, Production, and Uses.

[24] Stahl P., Lev Mirom Y., Stern R.A., Goldway M. (2019). Comparing ‘Iriet’ and ‘Ettinger’ Avocado Cultivars as Pollinators of ‘Hass’ Using SNPs for Paternal Identification. Sci. Hortic..

[25] McConchie C.A., Batten D.J., Vivian-Smith A. (1991). Pollination in Lychee. Yearb. Aust. Lychee Grow. Assoc..

[26] Xie D.-R., Ma X.-S., Rahman M.Z., Yang M.-C., Huang X.-M., Li J.-G., Wang H.-C. (2019). Thermo-Sensitive Sterility and Self-Sterility Underlie the Partial Seed Abortion Phenotype of Litchi Chinensis. Sci. Hortic..

[27] Jiang S.-Y., Xu H.-Y., Wang H.-C., Hu G., Li J.-G., Chen H.-B., Huang X. (2012). A Comparison of the Costs of Flowering in ‘Feizixiao’ and ‘Baitangying’ Litchi. Sci. Hortic..

[28] Sedgley M., Griffin A.R. (1989). Sexual Reproduction of Tree Crops.

[29] Wang J., Chen J., Huang S., Han D., Li J., Guo D. (2022). Investigating the Mechanism of Unilateral Cross Incompatibility in Longan (*Dimocarpus longan* Lour.) Cultivars (Yiduo × Shixia). Front. Plant Sci..

[30] Cuveas J., Polito V.S. (1997). Compatibility Relationships in ‘Manzanillo’ Olive. Hortic. Res..

[31] Sapir G., Stern R.A., Shafir S., Goldway M. (2008). S-RNase Based S-Genotyping of Japanese Plum (*Prunus salicina* Lindl.) and its Implication on the Assortment of Cultivar-Couples in the Orchard. Sci. Hortic..

[32] Goldway M., Stern R.A., Zisovich A.H., Shafir S. (2005). Application of S -RNase Allele Molecular Analysis as a Means for Elucidating Low Yields in the Pear Orchard. Acta Hortic..

[33] Zisovich A.H., Stern R.A., Shafir S., Goldway M. (2004). Identification of Seven S-Alleles from the European Pear (*Pyrus communis*)and the Determination of Compatibility among Cultivars. J. Hortic. Sci. Biotechnol..

[34] Schneider D., Stern R.A., Goldway M. (2005). A Comparison between Semi- and Fully Compatible Apple Pollinators Grown under Suboptimal Pollination Conditions. HortScience.

[35] Hajari E., Nonyane D., Cronje R. (2020). Sequence-Related Amplified Polymorphism Markers—A Tool for Litchi Breeders in Africa. S. Afr. J. Sci..

[36] Pathak A.K., Singh S.P., Tuli R. (2014). Amplified Fragment Length Polymorphism Fingerprinting to Identify Genetic Relatedness among Lychee Cultivars and Markers Associated with Small-Seeded Cultivars. J. Am. Soc. Hortic. Sci..

[37] Tongpamnak P., Patanatara A., Srinives P., Babprasert C. (2002). Determination of Genetic Diversity and Relationships among Thai Litchi Accessions by RAPD and AFLP Markers. Kasetsart J. Nat. Sci..

